# A comparison of progesterone via vaginal oil capsules versus pessaries for luteal phase support in assisted reproduction treatment: a multicentre cohort study of 42 291 cycles

**DOI:** 10.1093/humrep/deaf219

**Published:** 2025-11-21

**Authors:** R K Dhillon-Smith, M Khairy, T Bamford, V Sephton, A Richardson, A H Balen, A Coomarasamy

**Affiliations:** Department of Metabolism and Systems Science, School of Medical Sciences, Tommy’s National Centre for Miscarriage Research, University of Birmingham, Edgbaston, UK; National Institute for Health and Care Research (NIHR) Birmingham Biomedical Research Centre, Birmingham, UK; Care Fertility Birmingham, Birmingham, UK; Care Fertility Manchester, Manchester, UK; Care Fertility Group, Nottingham, UK; Care Fertility Northampton, Northampton, UK; Leeds Centre for Reproductive Medicine, Leeds Teaching Hospitals NHS Trust, Leeds, UK; Department of Metabolism and Systems Science, School of Medical Sciences, Tommy’s National Centre for Miscarriage Research, University of Birmingham, Edgbaston, UK; Nuffield Department of Women’s and Reproductive Health, University of Oxford, Oxford, UK

**Keywords:** progesterone, luteal phase support, ART, FET, frozen embryo transfer, miscarriage

## Abstract

**STUDY QUESTION:**

What is the effect of progesterone administered via vaginal oil capsules versus pessaries, on clinical outcomes, when used for luteal phase support (LPS) in ART?

**SUMMARY ANSWER:**

Our study findings indicate a higher live birth rate with vaginal oil capsules compared with pessaries, in both fresh and frozen cycles; in the frozen cycles, a lower miscarriage rate was observed with vaginal oil capsules compared with pessaries.

**WHAT IS KNOWN ALREADY:**

Sufficient LPS, with exogenous progesterone, is essential during ART to improve implantation and pregnancy rates. Micronized vaginal progesterone (MVP) is the most commonly used form of luteal support worldwide. There are no head-to-head comparisons of vaginal oil capsules versus pessaries, with a focus on clinical efficacy, for LPS.

**STUDY DESIGN, SIZE, DURATION:**

Retrospective cohort study of patients who completed ART cycles with either only vaginal oil capsules 600–800 mg/day or only pessaries 800 mg/day for LPS. Primary outcomes were live birth and miscarriage. Data for fresh IVF/ICSI cycles and frozen embryo transfer cycles with hormone replacement therapy (HRT-FET) were analysed separately. Multivariable regression analyses were performed with adjustment for female age, BMI, ethnicity, ovarian reserve, duration and cause of subfertility, stimulation protocol, number of previous cycles, number of oocytes, number of embryos transferred, previous live births, and previous miscarriages.

**PARTICIPANTS/MATERIALS, SETTING, METHODS:**

Our study population consisted of women undergoing treatment across 14 Care Fertility clinics in the UK, from January 2017 to December 2022. We included women with stimulated IVF/ICSI cycles with fresh embryo transfer and autologous HRT-FET cycles. A total of 42 291 cycles were analysed; vaginal oil capsules were exclusively used in 25 738 cycles and pessaries exclusively in 16 553 cycles.

**MAIN RESULTS AND THE ROLE OF CHANCE:**

In the IVF/ICSI group, the live birth rate was higher in those taking vaginal oil capsules compared with pessaries: 34.3% vs 27.8%; adjusted risk ratio (aRR) 1.11 (95% CI 1.04–1.19; *P* < 0.001). In the HRT-FET group, the live birth rate was also higher in those taking vaginal oil capsules compared to pessaries: 36.7% vs 32.9% (aRR 1.09; 95% CI 1.04–1.14; *P* < 0.001). The miscarriage rate was lower in those taking vaginal oil capsules compared to pessaries for both IVF/ICSI (13.4% vs 14.5%, *P* < 0.05) and HRT-FET cycles (17.2% vs 19.7%, *P* < 0.001) in the crude analysis. The adjusted analysis for miscarriage found a statistically significant difference only for HRT-FET cycles (aRR 0.87; 95% CI 0.82–0.93).

**LIMITATIONS, REASONS FOR CAUTION:**

This is a retrospective cohort study. Whilst we have extensively adjusted for confounding, there can still be residual confounding.

**WIDER IMPLICATIONS OF THE FINDINGS:**

An appropriately powered randomized controlled trial directly comparing the two drugs, focusing on clinical efficacy, is required to determine if one is superior to the other.

**STUDY FUNDING/COMPETING INTEREST(S):**

This study has been delivered through the National Institute for Health and Care Research (NIHR) Birmingham Biomedical Research Centre (BRC). R.K.D.S. is in receipt of honoraria for lectures and presentations from Ferring Pharmaceuticals and Besins Healthcare UK. R.K.D.S. has received travel support from IBSA Pharma and Theramex UK and payment for participation on an advisory board for educational meetings for Ferring Pharmaceuticals and IBSA Pharma. A.C. has contributed to the scientific advisory boards of Ferring Pharmaceuticals, Theramax UK, Besins Healthcare UK, and Organon Pharma UK. M.K. has received travel support from Besins Healthcare UK and Merck. A.H.B. is on an advisory board and has received speaker fees from NovoNordisk Pharmaceuticals and is a shareholder at Care Fertility UK and Care Fertility Leeds. T.B. is in receipt of honoraria for lectures and presentations from Merck and Gedeon Richter. T.B. has received travel support from IBSA Pharma, Vitrolife, and Theramex and payment for participation on an advisory basis for IBSA Pharma and Conceivable Life Sciences. A.R. has received honoraria for lectures and presentations from Gedeon Richter. A.R. has received travel support from Gedeon Richter and payment for participation on an advisory board for educational meetings for Ferring Pharmaceuticals. V.S. has received travel support from IBSA Pharma and payment for educational meetings and participation in an advisory role for Theramex.

**TRIAL REGISTRATION NUMBER:**

n/a

## Introduction

Luteal phase support (LPS) is an essential element in ART. This applies to both fresh IVF/ICSI cycles, and frozen embryo transfer cycles where hormone replacement therapy is used (HRT-FET). Stimulated ART cycles (IVF/ICSI) are known to have an insufficient luteal phase, resulting in insufficient progesterone production. This is most likely due to the supra-physiological oestrogen levels in the follicular phase, as a result of ovarian stimulation used to prepare for oocyte retrieval (Fatemi *et al.*, 2007). In an HRT-FET treatment cycle, there is a complete absence of endogenous progesterone production due to the cessation of the ovulation process and a lack of a corpus luteum. Therefore, sufficient LPS with exogenous progesterone is crucial during both fresh IVF/ICSI and HRT-FET cycles to improve implantation and pregnancy rates.

Progesterone for LPS in ART can be administered via different routes, including vaginal, rectal, oral, subcutaneous, or intramuscular injection. The dosing of progesterone and route of administration vary according to patient and doctor preferences (Vaisbuch *et al.*, 2014), although micronized vaginal progesterone (MVP) is the most used form worldwide (Di Guardo *et al.*, 2020). A web-based survey of data from 303 IVF units worldwide showed that vaginal administration was the predominant delivery route for progesterone; its use had risen from 64% of cycles in 2009 to 74% in 2019 (Shoham *et al.*, 2021).

Two of the most commonly used forms of MVP used for luteal support in the UK are a vaginal oil-based capsule (brand name, Utrogestan^®^) and a pessary (brand name, Cyclogest^®^). Utrogestan^®^ is administered vaginally at a dose of 200 mg three times daily or 400 mg twice daily, and Cyclogest^®^ can be administered vaginally or rectally at a dose of 400 mg twice daily. Alternative MVP options are vaginal tablets (Lutigest^®^) and vaginal gel (Crinone^®^). A systematic review of randomized controlled trials found no differences in efficacy or safety amongst any of the different MVP formulations, although the quantity and quality of the available evidence was limited (Child *et al.*, 2018). Data from trials also show that both Cyclogest^®^ and Utrogestan^®^ are acceptable and tolerable to patients (Kleinstein and Luteal Phase Study Group, 2005; Saunders *et al.*, 2020).

There are no high-quality data or appropriately powered randomized trials, focusing on clinical efficacy, on head-to-head comparisons of Cyclogest^®^ versus Utrogestan^®^. The choice of which to use is down to individual clinic preference or clinician discretion, while cost is also an influencing factor. The aim of this study was to focus on the clinical efficacy of Utrogestan^®^ versus Cyclogest^®^ in women undergoing either fresh IVF/ICSI or HRT-FET treatment cycles.

## Materials and methods

### Study design

This is a retrospective cohort study which included all women undergoing autologous cycles of IVF, ICSI, or frozen embryo transfer with hormone replacement treatment (HRT-FET) at Care Fertility group in the UK and Ireland, from January 2017 to December 2022. Care Fertility is one of the UK’s largest independent providers of fertility services. The study period was selected to allow for inclusion of complete live birth data and because serum progesterone testing, and subsequent luteal phase ‘rescue treatment’, was not routine practice at the time. This allowed for a cleaner head-to-head comparison of the two drugs used exclusively for LPS.

### Inclusion criteria

We included all stimulated IVF/ICSI and frozen embryo transfer cycles in the study period in which there was use of either Utrogestan^©^ or Cyclogest^©^ vaginal pessaries as the sole medication for LPS.

### Exclusion criteria

We excluded women who had ovulation induction or intrauterine insemination cycles, oocyte donors, and surrogate hosts. We also excluded women who had either subcutaneous progesterone or any additional forms of progesterone in combination with Utrogestan^©^ or Cyclogest^©^.

### Ethics

Permission to utilize an anonymized dataset of the routinely collected electronic data was granted by the Institutional Review Board of Care Fertility. As the study utilized routine clinical data with no novel intervention, external ethics committee approval was not sought as per the clinical decision tool of the Health Research Authority (HRA) in the UK.

### Cycle protocols

The stimulated IVF/ICSI cycles were performed with either a long agonist protocol or short antagonist protocol. Pituitary suppression was achieved, in the long protocol, with GnRH agonist in the form of either Buserelin 0.5 ml (Supercur^©^, Sanofi Aventis UK) subcutaneously daily, or Nafarelin 400 mcg (Synarel^©^, Pfizer UK) nasal spray 2 puffs daily, from Day 21 of the preceding cycle and throughout ovarian stimulation. In the short protocol, GnRH antagonist, Cetrorelix 0.25 mg (Cetrotide^©^, Merck Serono), or Ganirelix 0.25 mg (Orgalutran^©^ or Ganirelix^©^, Gideon Richter) was given by subcutaneous injection daily starting from Day 6 of ovarian stimulation. Ovarian stimulation was achieved with the use of either human menopausal gonadotropin (Menopur^©^, Ferring UK, or Meriofert^©^, IBSA) or recombinant FSH (Gonal F^©^, Merck Serono) or combination therapy in doses ranging from 150 to 450 IU daily. The dose was selected according to the woman’s individual clinical profile, taking into account age, BMI, anti-Müllerian hormone (AMH), and previous response to ovarian stimulation. The gonadotropins were initiated after confirmation of pituitary downregulation in the long protocol or on cycle days 1–2 after start of menses in the antagonist protocol and continued till administration of hCG (Gonasi^©^ 10 000 IU, IBSA or Ovitrelle^©^ 250 µg, Merck Sorono). In cases where the patient was considered to be at risk of ovarian hyperstimulation syndrome, where appropriate, a GnRH agonist was administered in the form of Buserelin 0.1 ml or Decapeptyl 0.2 ml as the drug for ovulatory trigger before oocyte retrieval 36 h later.

The luteal support in the stimulated cycle was initiated on the evening of the day of oocyte retrieval with either Utrogestan^®^ (Besins, Belgium) administered vaginally at a dose of 200 mg vaginal pessaries three times a day or Cyclogest^©^ (L D Collins, UK) 400 mg vaginal pessaries twice daily. The choice of either Utrogestan^©^ or Cyclogest^©^ was primarily by the care-providing clinician’s discretion, although the choice was by patients’ preference in some cases.

In the frozen embryo transfer cycles, patients had endometrial preparation with oral oestradiol at a dose of 6–12 mg/day. For patients at high risk of thromboembolism, 100–200 mcg patches changed every 3 days were given instead of oral oestradiol. The dosing was based on the patients’ BMI and previous endometrial thickness in stimulated or FET cycles. Following ultrasound confirmation of an adequate endometrial thickness (>7 mm), LPS was commenced with vaginal progesterone in the form of either Utrogestan^©^, at a dose of 400 mg (2 × 200 mg vaginal capsules) twice daily, or Cyclogest^®^ at a dose of 400 mg (one 400 mg pessary) twice daily administered vaginally. During the study period, there was no routine measurement of serum progesterone levels in the FET cycles and thus no luteal phase ‘rescue’ therapy protocol existed.

### Outcome measures

The primary outcomes of the study were live birth rate (LBR) per cycle and miscarriage rate (MR) per cycle, with clinical pregnancy rate (CPR) as a key secondary outcome. Live birth was defined as any pregnancy resulting in the delivery of a live foetus after 24 weeks of gestation. The outcome was the number of live birth events (singleton or multiple), i.e. for each cycle of treatment, there was either one outcome of live birth or not. Clinical pregnancy was defined as any viable pregnancy with demonstrable foetal heart pulsations on transvaginal ultrasound. Miscarriage was defined as the loss of a biochemical or clinical pregnancy. We further categorized miscarriage into ‘early’ and ‘late’. Early miscarriage was defined as any pregnancy loss after the detection of a positive urine or serum hCG test and before the detection of a gestational sac on transvaginal ultrasound at the viability scan performed at 7 weeks’ gestation. Late (clinical) miscarriage was defined as any pregnancy loss after the detection of an intrauterine gestational sac by transvaginal ultrasound at the viability scan at 7 weeks’ gestation.

### Study analysis plan

Comparison of baseline clinical and laboratory characteristics of all included cycles, by the type of luteal regimen, was planned. It was decided *a priori* that the data for stimulated IVF/ICSI and FET cycles would be analysed separately for primary and secondary outcomes. In addition, the regression analyses of primary and secondary outcomes would be conducted in these two groups separately. In the multivariable regression analysis for stimulated IVF/ICSI cycles, the impact of luteal support regimen (Cyclogest^©^ as reference medication vs Utrogestan^©^) was assessed after adjustment for the following 12 covariates: female age, BMI, duration of subfertility, number of previous ART cycles, AMH as an indicator of ovarian reserve, number of oocytes retrieved, number of embryos transferred, different aetiologies of subfertility (male, ovulatory, tubal, uterine and unexplained), type of stimulation protocol (agonist protocol vs antagonist), ethnicity, previous live births achieved and previous miscarriages. The latter two variables were used as categorical variables. The covariates were selected either due to their known effect on IVF outcome or if found to be statistically different between the groups in baseline comparisons or in univariate regression analyses. In the regression analysis for FET cycles, adjustment was made for the same covariates above, with the exception of AMH, number of oocytes retrieved, and stimulation protocol (a total of nine covariates). For all regression analyses, we conducted logistic regression analyses with estimation of adjusted odds ratio (aOR); however, we also conducted modified Poisson regression analyses with robust estimation of error function to report on adjusted risk ratio (aRR). We decided to report the final findings using the aRR as it has less risk of overestimation of treatment effect on outcomes when the outcomes are relatively common (outcome prevalence >10%).

Multivariable regression analyses, to test the interaction between the luteal support regimen and BMI, categorical age groups, and number of previous ART cycles, for the outcome of live birth, were performed. *A priori* subgroup analyses were planned in treatment-naive patients having their first ART cycle, with the same adjustment for confounders as detailed above. Additionally, we conducted regression analyses in patients with singleton pregnancies to exclude the impact of multiple pregnancy on pregnancy outcome. To address the issue of missing data, we performed multiple imputation analyses for the outcomes of clinical pregnancy, miscarriage, and live birth, and any covariates with >10% missing data. Finally, we calculated the predicted mean probability of live birth in each age category and in each ethnic group by type of luteal support used, and this was with adjustment for other confounders. Maternal age was selected as this is the strongest predictor for live birth, and ethnicity was selected as it is well established that there are significant ethnic variations in ART success rates.

### Statistical analysis

The baseline patient characteristics, cycle characteristics, and outcome data were described, giving frequencies with percentages, or medians with interquartile range (IQR), as appropriate. Comparison of continuous variables was performed with non-parametric tests (Mann–Whitney *U* test) as assumptions of normality could not be fulfilled. For categorical variables, we used the chi-square test. The enter method was used in the multivariable regression analysis, as the power of the study sample was suitable for inclusion and adjustment for all potential predictors and confounders simultaneously to have more precise and robust estimates of impact of LPS medication on outcome and to avoid an overfitted model. We also checked for multicollinearity, heteroskedasticity, and performed goodness-of-fit tests for the fitted regression models. In the logistic regression models, there was no evidence of multicollinearity, as all predictors had tolerance values (1/VIF) above the cut-off of 0.2. In addition, the non-significant Hosmer–Lemeshow test and acceptable overall model accuracy, as indicated by the likelihood ratio test, supported the model’s adequacy. For the Poisson regression models, the goodness-of-fit test showed acceptable fitting and no evidence of dispersion.

A *P*-value of less than 0.05 was considered as the cut-off for statistical significance. For all regression analyses, propensity score matching was performed, and the covariates adjusted for were consistent across the IVF/ICSI cycles (12 covariates) and HRT-FET (9 covariates) cycles. All analyses were performed using STATA 17 (STATA Corp., Texas, USA).

## Results

A total of 42 291 cycles in 23 149 women were found to be eligible for inclusion during the study period. These cycles were conducted in 14 assisted conception units across the UK, which are part of the Care Fertility group. Cyclogest^©^ was used in 16 553 cycles and Utrogestan^©^ in 25 738 cycles. Live birth outcome data were available in 17 923 IVF/ICSI cycles and 18 209 HRT-FET cycles.

### Baseline and cycle characteristics

Comparison of baseline clinical characteristics between Cyclogest^©^ and Utrogestan^©^ is given in Table 1. Women in the Cyclogest^©^ group were significantly older than the women in the Utrogestan^©^ group, with a median age of 36 years (IQR 33–40) in the Cyclogest^©^ group as compared to a median age of 35 (IQR 32–39) in the Utrogestan^©^ group. There was a significant difference in ovarian reserve between the two groups, with a lower median AMH (14.5 vs 15.2 pmol/l), lower antral follicle count (13 vs 16), and a higher FSH (6.7 vs 6.4 IU/L) in the Cyclogest^©^ group compared with the Utrogestan^©^ group. On the other hand, there was a statistically significantly higher median BMI in the Utrogestan^©^ arm, compared with the Cyclogest^©^ arm (24.7 vs 23.9 kg/m^2^, *P* < 0.001). Differences in the distribution of IVF, ICSI, and FET cycles and other surrogate cycle outcomes, such as the number of oocytes, number of embryos, and number of embryos transferred between the Cyclogest^©^ and Utrogestan^©^ groups, are shown in Table 2.

**Table 1.** Comparisons of baseline clinical characteristics.

	**Cyclogest** ^©^	**Utrogestan** ^©^	*P*-value
	(n = 16553)	(n = 25738)	
Age (in years)	36 (33–40)	35 (32–39)	<0.001
Median (IQR)			
BMI (kg/m^2^)	23.9 (21.6–27)	24.7 (22–28.2)	<0.001
Median (IQR)			
Ethnicity			
White	9910 (59.9%)	19 343 (75.2%)	0.02
Asian	1608 (9.7%)	2987 (11.6%)	0.3
Black	279 (1.7%)	499 (1.9%)	0.001
Chinese	162 (1%)	266 (1.03%)	<0.001
Mixed/other	529 (3.2%)	734 (2.9%)	<0.001
Cause of infertility			
Ovulatory	582 (3.5%)	2065 (8%)	<0.001
Tubal	755 (4.6%)	2039 (7.9%)	<0.001
Uterine or peritoneal	779 (4.7%)	1720 (6.7%)	<0.001
Male factor	1131 (6.8%)	3739 (14.5%)	<0.001
Unexplained	6109 (36.9%)	5552 (21.6%)	<0.001
Mixed	7197 (43.5%)	10 623 (41.3%)	<0.01
Previous live birth	3530 (21.4%)	5609 (21.8%)	0.7
Previous miscarriage^#^	1694 (10.2%)	2719 (10.7%)	0.3
Previous recurrent miscarriages^‡^	662 (4%)	888 (3.5%)	<0.01
Duration of infertility (years)	2.2 (1.5–3)	2.4 (2–3.6)	0.7
Median (IQR)			
Number of ART cycles	1 (1–2)	2 (1–3)	<0.01
Median (IQR)			
Day 2 FSH (IU/L)	6.7 (5.5–8.8)	6.4 (5.1–8)	0.28
Median (IQR)			
AMH level (pmol/L)	14.5 (7.7–25.4)	15.2 (7.8–26.8)	<0.01
Median (IQR)			
Antral follicle count	13 (6–21)	16 (9–26)	<0.001
Median (IQR)			

IQR = interquartile range; AMH = anti-Mullerian hormone.

# Previous miscarriages include all women with at least one previous miscarriage.

‡ Recurrent miscarriages were defined as cases with two or more previous miscarriages.

*P*-value < 0.05 was considered significant.

**Table 2.** Comparison of cycle outcome data.

	**Cyclogest** ^©^ **(n = 16553)**	**Utrogestan** ^©^ **(n = 25738)**	*P*-value
Treatment type			
IVF	4234 (25.6%)	5687 (22.1%)	<0.001
ICSI	4689 (28.3%)	9663 (37.5%)	<0.001
FET	7630 (46%)	10 388 (40.4%)	<0.001
Number of oocytes retrieved			
Median (IQR)	9 (5–14)	9 (6–14)	<0.001
Total number of embryos			
Median (IQR)	4 (2–7)	5 (3–8)	<0.01
Number of embryos transferred			
Median (IQR)			
IVF/ICSI	1 (0–1)	1 (0–1)	<0.01
FET	1 (1–1)	1 (1–1)	<0.05
Fertilization rate (%)			
Median (IQR)	61.7 (44.4–76)	60 (44–75)	0.26
ICSI	60 (41.7–75)	58 (42.8–75)	0.99
IVF	65 (50–80)	66 (50–80)	0.31
Number of embryos frozen			
Median (IQR)	1 (0–3)	1 (0–3)	0.56
Endometrial thickness			
Median (IQR)			
IVF/ICSI	10.5 (9–12)	11 (9.4–12.6)	<0.001
FET	9 (8.2–10.2)	9.1 (8.2–10.4)	<0.01

FET = frozen embryo transfer; IQR = interquartile range.

*P*-value <0.05 was considered significant.

Despite the majority of patients having an elective single embryo transfer, we still found a significant difference between the groups, due to the absence of an embryo transfer in some stimulated cycles. Furthermore, whilst the number of absence of embryo transfers was smaller in HRT-FET cycles, as reflected by IQR of (1–1), there was still a statistically significant difference between the groups due to the small number of patients who either had no embryos transferred or had two embryos transferred, hence we have included the number of embryos transferred in our regression models.

### Clinical outcomes

The outcome data for clinical pregnancy, miscarriage, and LBR are presented in Table 3 (IVF/ICSI cycles) and Table 4 (HRT-FET cycles). The unadjusted and adjusted relative risks for the outcomes of miscarriage, clinical pregnancy, and live birth, for both fresh and frozen cycles, are presented in Table 5. The full regression analysis models are presented in Supplementary Tables S1, S2, and S3. For completeness, the results with the odds ratios (unadjusted and adjusted) are presented in Supplementary Table S4.

**Table 3.** Clinical outcome data for stimulated IVF/ICSI cycles with fresh embryo transfer in the two luteal support groups.

	**Cyclogest** ^©^	**Utrogestan** ^©^	*P*-value
	(n = 6170)	(n = 11753)	
Pregnancy rate	2614 (42.4%)	5600 (47.6%)	<0.0001
Clinical pregnancy rate*	2006 (32.5%)	4524 (38.5%)	<0.0001
Pregnancy outcome			
Live birth^a^	1715 (27.8%)	4026 (34.3%)	<0.0001
Early miscarriage^b^	607 (9.8%)	1075 (9.2%)	0.13
Late miscarriage^c^	291 (4.7%)	498 (4.2%)	0.14
Total miscarriage	898 (14.5%)	1573 (13.4%)	<0.05

*Defined as the presence of a gestational sac by ultrasound during the first trimester.

a Expressed as a percentage of all cycles.

b Early miscarriage is defined as biochemical loss (no pregnancy seen on scan)—expressed as a percentage of biochemical pregnancies.

c Late miscarriage is defined as clinical pregnancy confirmed on scan at 7 weeks with no fetal heartbeat (FH) at booking scan—expressed as a percentage of clinical pregnancies.

**Table 4.** Clinical outcome data for HRT-FET cycles in the two luteal support groups.

	**Cyclogest** ^©^ **(n = 7630)**	**Utrogestan** ^©^ **(n = 10579)**	*P*-value
Biochemical pregnancy rate	4005 (52.5)	5697 (53.9%)	0.07
Clinical pregnancy rate*	2875 (37.7%)	4327 (40.9%)	<0.0001
Pregnancy outcome			
Live birth^a^	2507 (32.9%)	3879 (36.7%)	<0.0001
Early miscarriage^b^	1128 (14.8%)	1368 (12.9%)	<0.001
Late miscarriage^c^	372 (4.9%)	449 (4.3%)	<0.05
Total miscarriage	1500 (19.7%)	1817 (17.2%)	<0.001

*Defined as the presence of a gestational sac by ultrasound during the first trimester.

a Expressed as a percentage of all cycles.

b Early miscarriage is defined as biochemical loss (no pregnancy seen on scan)—expressed as a percentage of biochemical pregnancies.

c Late miscarriage is defined as clinical pregnancy confirmed on scan at 7 weeks with no FH at booking scan—expressed as a percentage of clinical pregnancies.

**Table 5.** Univariate and multivariate regression analyses for pregnancy outcomes in all cycles.

	Univariate regression	Multivariate regression
	RR (95% CI)	Adjusted RR (95% CI)
Clinical pregnancy		
IVF/ICSI cycles	1.18 (1.13–1.24)	1.08 (1.02–1.15)^‡^
HRT-FET cycles	1.09 (1.05–1.13)	1.07 (1.02–1.11)^#^
Total miscarriage		
IVF/ICSI cycles	0.92 (0.83–0.99)	0.99 (0.89–1.12)^‡^
HRT-FET cycles	0.87 (0.82–0.93)	0.87 (0.82–0.93)^#^
Early miscarriage		
IVF/ICSI cycles	0.93 (0.84–1.02)	1.05 (0.91–1.21)^‡^
HRT-FET cycles	0.87 (0.81–0.94)	0.87 (0.80–0.94)^#^
Late miscarriage		
IVF/ICSI cycles	0.89 (0.77–1.03)	0.91 (0.74–1.12)^‡^
HRT-FET cycles	0.87 (0.76–0.99)	0.89 (0.77–1.02)^#^
Live birth		
IVF/ICSI cycles	1.23 (1.18–1.29)	1.11 (1.04–1.19)^‡^
HRT-FET cycles	1.12 (1.07–1.16)	1.09 (1.04–1.14)^#^

RR = relative risk; HRT-FET = hormone replacement therapy-frozen embryo transfer.

Cyclogest^©^ was used as the reference group.

‡ Logistic regression model adjusting for 12 covariates in IVF/ICSI cycles (age of female partner, BMI, AMH, number of oocytes, number of embryos transferred, cause of subfertility, ethnicity, protocol of ovarian stimulation, duration of subfertility, number of previous ART cycles, previous livebirths, and previous miscarriages).

# Logistic regression model adjusting for 9 covariates in HRT-FET cycles (age of female partner, BMI, number of embryos transferred, cause of subfertility, ethnicity, duration of subfertility, number of previous ART cycles, previous livebirths, and previous miscarriages).

#### Clinical pregnancy

In the IVF/ICSI cycles, there was a significantly higher CPR in the Utrogestan^©^ group compared to Cyclogest^©^ (38.5% vs 32.5%, *P* < 0.0001) (Table 3). Similar results were observed for the HRT-FET cycles (Table 4), with a higher CPR in the Utrogestan^©^ group vs the Cyclogest^©^ (40.9% vs 37.7%, *P* < 0.0001). Multivariable regression analyses, after controlling for covariates and conducting propensity score matching, showed a higher rate of clinical pregnancy in both the IVF/ICSI and HRT-FET cycles for the Utrogestan^©^ group compared to the Cyclogest^©^ group: aRR 1.08 (95% CI 1.02–1.15) and aOR 1.07 (95% CI 1.02–1.11), respectively (Table 5).

#### Miscarriage

In the IVF/ICSI cycles, there was a significantly lower MR in the Utrogestan^©^ group compared to Cyclogest^©^ (13.4% vs 14.5%, *P* < 0.05) (Table 3). Similar findings were also detected for the HRT-FET cycles (Table 4) with lower MRs observed in the Utrogestan^©^ group (17.2% vs 19.7%, *P* < 0.001). Multivariable regression analyses, after controlling for covariates and conducting propensity score matching, showed a significant reduction in the rate of total miscarriages in the HRT-FET cycles with Utrogestan use (aRR 0.87 (95% CI 0.82–0.93), Table 5). However, there was no difference seen in the adjusted analyses for MR in the IVF/ICSI cycles (Table 5).

#### Live birth (LBR)

There was a significantly higher LBR (34.3% vs 27.8%, *P* < 0001) in the Utrogestan^©^ group compared to the Cyclogest^©^ group for the fresh IVF/ICSI cycles (Table 3). Similar findings were shown for the HRT-FET cycles with a higher LBR (36.7% vs 32.9%, *P* < 0.0001, Table 4) in the Utrogestan^©^ group. Multivariable regression analyses, after controlling for the covariates and conducting propensity score matching, showed a higher rate of live birth in both the IVF/ICSI and HRT-FET cycles for the Utrogestan^©^ group: aRR 1.11 (95% CI 1.04–1.19) and aRR 1.09 (95% CI 1.04–1.14), respectively (Table 5).

**Figure 1.**
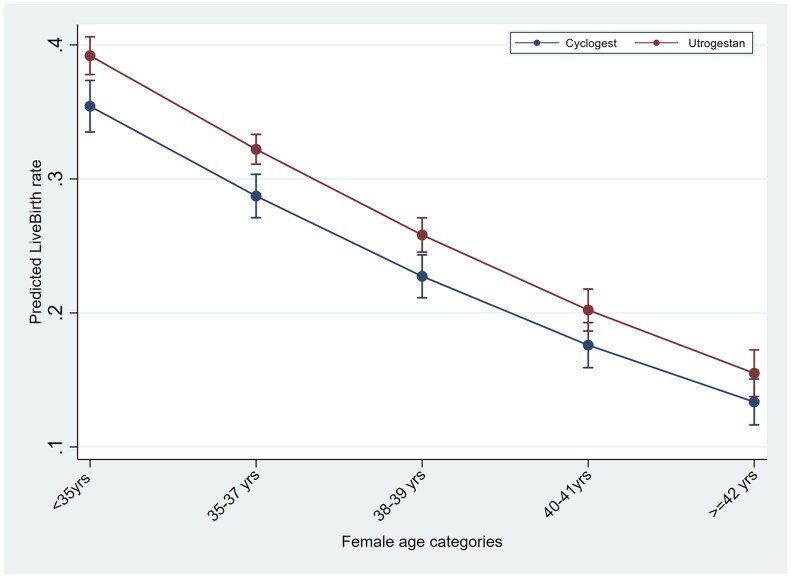
Predicted live birth by age categories and luteal support in IVF/ICSI cycles.

**Figure 2.**
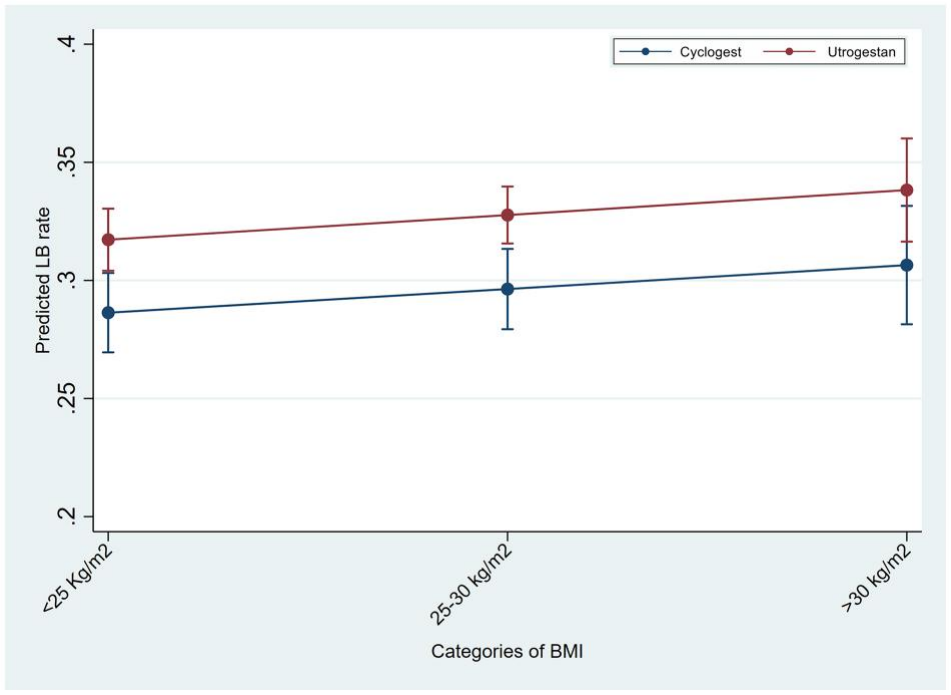
Predicted live birth by BMI categories and luteal support in IVF/ICSI cycles.

**Figure 3.**
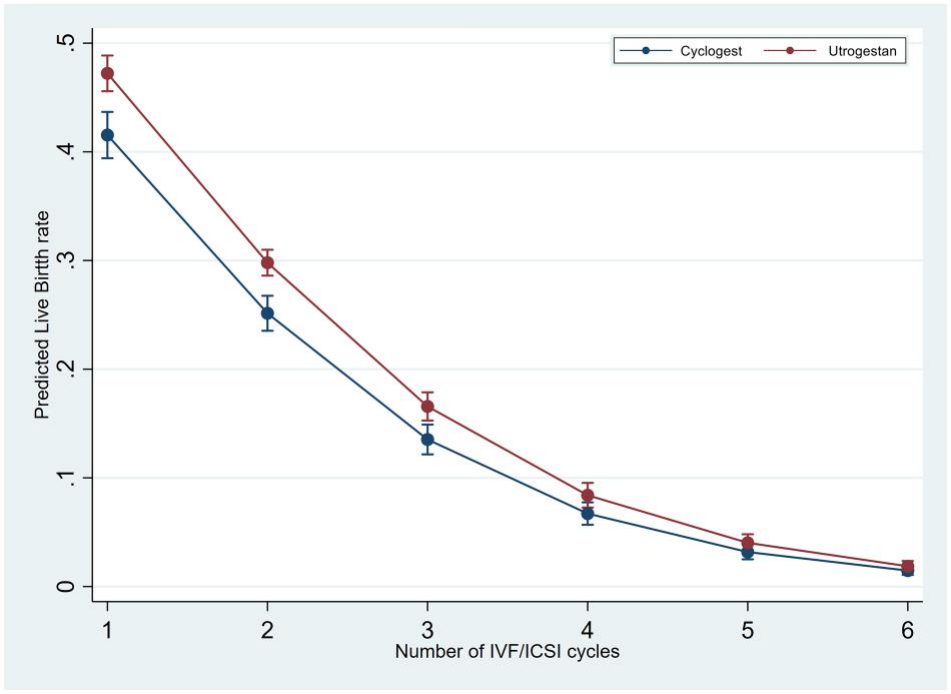
Predicted live birth by number of cycles and luteal support in IVF/ICSI cycles.

**Figure 4.**
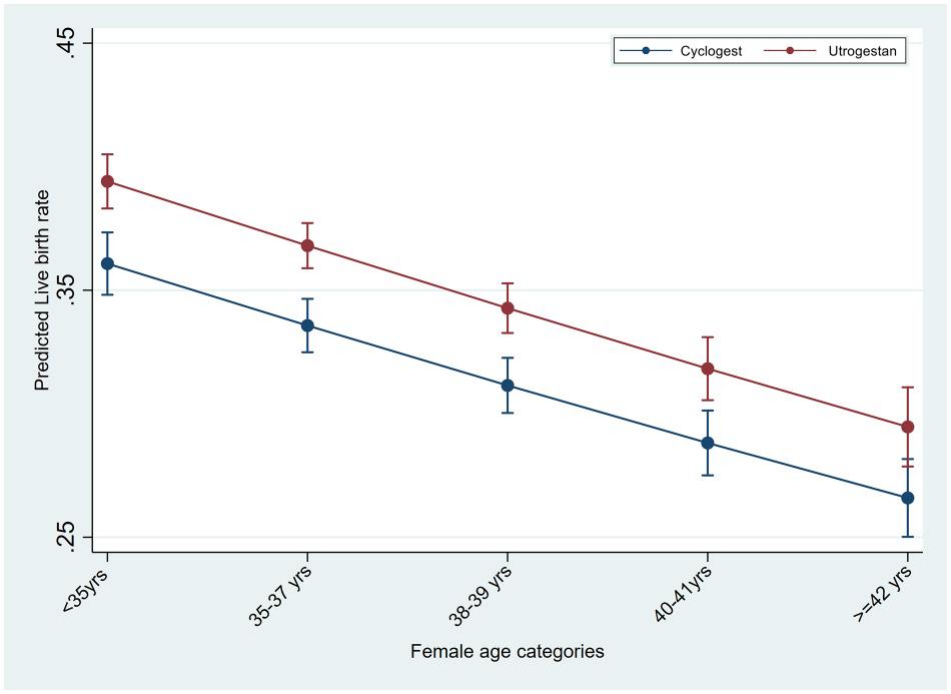
**Predicted live birth by age categories and luteal support in HRT-FET cycles.** HRT-FET = hormone replacement therapy-frozen embryo transfer.

**Figure 5.**
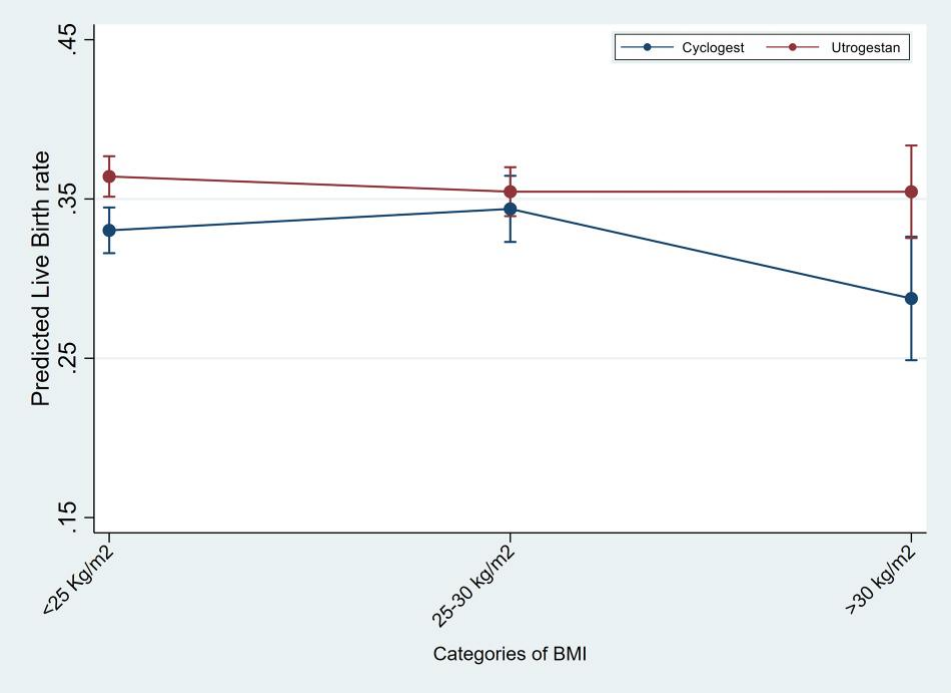
**Predicted live birth by BMI categories and luteal support in HRT-FET cycles.** HRT-FET = hormone replacement therapy-frozen embryo transfer.

**Figure 6.**
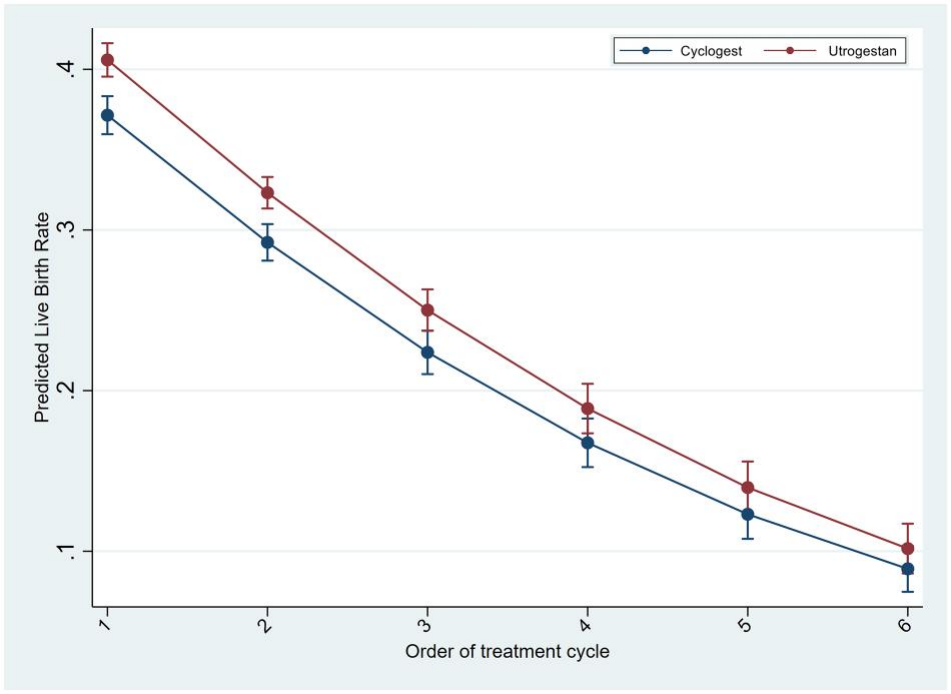
**Predicted live birth by number of cycles and luteal support in HRT-FET cycles**. HRT-FET = hormone replacement therapy-frozen embryo transfer.

In the logistic regression analyses for the outcome of live birth in IVF/ICSI cycles, testing the interaction of luteal support with age, BMI and number of previous cycles, showed significantly higher LBR in the Utrogestan^©^ group compared to Cyclogest^©^ in all age groups under 40 years, BMI under 30 kg/m^2^ and in the first three IVF/ICSI cycles (Figs 1, 2, and 3). For the HRT-FET cycles, tests for interaction showed significantly higher LBR in the Utrogestan^©^ group compared to Cyclogest^©^ in age groups under 42 years, BMI group <25 kg/m^2^ and in the first two HRT-FET cycles (Figs 4, 5, and 6). The mean predicted probability of live birth was higher with Utrogestan^©^ compared to Cyclogest^©^ in all age groups and across all ethnicities in all cycles (Supplementary Figs S1 and S2).

#### Additional analyses

Additional analyses were performed in women undergoing their first cycle of ART (Supplementary Table S5). This showed Utrogestan^©^ was, once again, associated with a higher rate of live birth compared to Cyclogest^©^ in both IVF/ICSI cycles (aRR 1.11; 95% CI 1.03–1.19) and in the HRT-FET cycles (aRR 1.15; 95% CI 1.07–1.24). Similarly, there was a higher CPR in women treated with Utrogestan^©^ in both IVF/ICSI cycles (aRR 1.08; 95% CI 1.01–1.15) and in the HRT-FET cycles (aRR 1.15; 95% CI 1.07–1.23). There was also a lower risk of miscarriage in the HRT-FET cycles (aRR 0.87; 95% CI 0.75–1.00), which was mainly due to a lower risk of early miscarriages (aRR 0.81; 95% CI 0.68–0.95) (Supplementary Table S5).

Further analyses were restricted to women having singleton pregnancies only (Supplementary Table S6). For the IVF/ICSI cycles, there was a higher clinical pregnancy (aRR 1.08; 95% CI 1.02–1.15) and LBR (aRR 1.11; 95% CI 1.04–1.19) in the Utrogestan^©^ group compared to Cyclogest^©^. For the HRT-FET cycles, there was also a higher clinical pregnancy (aRR 1.06; 95% CI 1.02–1.11) and live birth (aRR 1.09; 95% CI 1.04–1.14) and a lower risk of miscarriage (aRR 0.87; 95% CI 0.82–0.93) in the Utrogestan^©^ group compared to Cyclogest^©^.

Finally, regression analyses were performed after multiple imputations for missing data (Supplementary Table S7). This analysis found consistent results with Utrogestan^©^, once again, associated with a higher rate of live birth compared to Cyclogest^©^ in both IVF/ICSI cycles (aRR 1.05; 95% CI 1.01–1.10) and in the HRT-FET cycles (aRR 1.10; 95% CI 1.06–1.15) and lower risk of miscarriage in HRT-FET cycles (aRR 0.87; 95% CI 0.82–0.93). Higher chances of clinical pregnancy were observed in both IVF/ICSI (aRR 1.05; 95% CI 1.01–1.10) and HRT-FET cycles (aRR 1.08; 95% CI 1.04–1.12).

We performed an additional multi-level regression analysis, using ethnicity as a contextual level II variable, in view of previous data showing different success rates in different ethnicities. This showed results consistent with all other analyses, with an independent, significant, superior effect of Utrogestan^©^ (compared to Cyclogest^©^) on pregnancy outcomes after adjusting for potential variations related to ethnicity (Supplementary Table S8).

## Discussion

This was a large multicentre cohort study directly comparing the clinical outcomes in women undergoing fresh (IVF/ICSI) or frozen (HRT-FET) ART cycles, with either progesterone vaginal oil capsules (Utrogestan^®^) or progesterone pessaries (Cyclogest^®^) for LPS. Our results indicate improved CPRs and LBRs using Utrogestan^®^ compared to Cyclogest^®^ in all cycles, and reduced MRs specifically in frozen embryo transfer cycles. This study has found that for fresh IVF/ICSI cycles, there is an 8% increase in the chance of having a clinical pregnancy and an 11% increase in the chance of having a live birth with Utrogestan^®^ compared to Cyclogest^®^. For HRT-FET cycles, there is a 7% increased chance of having a clinical pregnancy, a 9% increased chance of having a live birth, and a 13% reduction in the chance of having a miscarriage when taking Utrogestan^®^ compared to Cyclogest^®^. We believe these are clinically meaningful differences in outcomes in a field where even fine margins of gain are strived for.

There were notable differences in baseline characteristics between the groups, with a higher number of older women and women with lower ovarian reserve in the Cyclogest^®^ group. However, these variables, along with others, were included in the multivariable regression analyses, where propensity score matching was performed, and the findings remained consistent, showing a higher CPR and LBR with Utrogestan^®^ compared to Cyclogest^®^.

Testing the interaction of luteal support with specific variables showed a significantly higher LBR in the Utrogestan^©^ group compared to Cyclogest^©^ in all age groups under 40 years, in the first three IVF/ICSI cycles, and the first two HRT-FET cycles.

### Serum progesterone levels: the debate

There is a general consensus, first suggested by Labarta et al., that there is a minimum clinically important serum luteal progesterone level, above which there are higher clinical pregnancy and LBRs in women undergoing FET (Labarta *et al.*, 2021). In a systematic review by Melo et al., this serum progesterone cut-off was found to be approximately 10 ng/ml (Melo *et al.*, 2021). Based on these studies, many fertility clinics worldwide have adopted the routine measurement of serum progesterone concentrations in FET cycles, in order to individualize the dosing for LPS. In cases where the serum progesterone is considered below the optimal threshold, a ‘rescue’ strategy is employed where the dosing is increased and/or alternative forms of progesterone are added. It is noteworthy that, in contrast to the suggestion of a cut-off of 10 ng/ml, a recent retrospective cohort study of 1016 cycles found that applying a cut-off of 5 ng/ml for serum progesterone levels appeared to be sufficient for the patient not to require additional ‘rescue’ progesterone, and resulted in comparable ongoing pregnancy rates (Vandierendonck *et al.*, 2024). There is less clarity over the value of serum progesterone testing for fresh embryo transfer cycles, with studies showing mixed findings (Rottiers *et al.*, 2024; Maignien *et al*., 2025).

A cohort study of 2665 HRT-FET cycles, by Labarta et al., compared Cyclogest^®^ versus Utrogestan^®^ using serum progesterone levels, taken on the day of embryo transfer, as the primary outcome (Labarta *et al.*, 2024). They found that patients using Cyclogest^®^ pessaries (at a dose of 400 mg BD) had significantly higher mean serum progesterone levels compared to women taking Utrogestan^®^ (also at a dose of 400 mg BD), with a lower risk of falling below the critical serum progesterone threshold. Consequently, there was a reduced need for a ‘rescue’ progesterone strategy in the Cyclogest^®^ group. This is in contrast to findings from a recent systematic review and meta-analysis, which found that the total daily dose of progesterone exerts a greater influence on the serum progesterone concentration than the product itself (Alsbjerg and Humaidan, 2025). There appears to be agreement amongst studies that for those patients undergoing HRT-FET cycles with a low serum progesterone concentration who are treated with supplementary progesterone, this improves their reproductive outcome (Álvarez *et al.*, 2021; Alsbjerg *et al.*, 2023; Stavridis *et al.*, 2023).

Despite a growing body of evidence to suggest various serum progesterone cut-offs below which adverse clinical outcomes occur, there is no strict correlation between progesterone plasma levels, tissue levels, histological pattern, and pharmacodynamic characteristics; this is partly due to the first uterine pass effect (Bourgain *et al.*, 1990; Miles *et al.*, 1994; Cicinelli *et al.*, 2000; Kolibianakis *et al.*, 2003). Therefore, the true relevance of applying serum progesterone levels as a sole surrogate marker for clinical outcomes, if they do not accurately represent the activity at the endometrial tissue level, needs careful exploration. For this reason, we purposefully focused our study on clinical efficacy. This is important as different vaginal preparations are likely to induce varied pharmacological effects, different adhesion to vaginal mucosa, unequal transport to the uterus, and specific endometrial progesterone levels. The differences in clinical outcomes observed in our study may be explained by pharmacodynamics and/or potential deciduogenic properties of the drugs. There is indirect evidence from pharmacodynamic studies comparing the AUC of plasma levels of progesterone after Utrogestan^®^ vs Crinone and Cyclogest^®^ vs Crinone; this evidence is suggestive of higher AUC levels and more stable progesterone levels following Utrogestan^®^ administration (Kleinstein and Luteal Phase Study Group, 2005; Duijkers *et al.*, 2018).

The serum progesterone concentration impacts reproductive outcomes in HRT-FET cycles significantly, as endometrial receptivity and pregnancy depend solely on the exogenous progesterone. Whilst there is undoubtedly a role for serum progesterone testing in HRT-FET cycles, a single serum snapshot result is unlikely to accurately represent the wider picture of progesterone absorption and activity in the endometrium. Furthermore, it is unlikely that a specific cut-off value exists in determining which women are at risk of poorer reproductive outcomes, when numerous factors affect ART outcomes. There are some patients for whom the cut-off of 10 ng/ml might, in fact, be too low. For example, women with adenomyosis and/or endometriosis are known to have abnormal oestrogen action in their endometrium, which results in progesterone resistance (Bulun *et al.*, 2023). It has been shown that a significantly higher threshold for optimal serum progesterone exists in patients with endometriosis, compared to those without endometriosis (Alsbjerg *et al.*, 2023).

A ‘one-size-fits-all’ approach using a cut-off of 8–10 ng/ml for serum progesterone levels is unlikely to be appropriate for all women. Many women will have successful pregnancies with lower levels of serum progesterone, whilst others (e.g. women with adenomyosis) will need much higher levels. It is likely that any clinical differences observed in the various progesterone formulations is based on bioavailability at a tissue level and dosing regimens, rather than based on serum progesterone levels. Dosing, in particular, is a key factor in determining outcomes from ART cycles. A recent systematic review of 11 014 patients found no significant differences between progesterone products with an equivalent daily dose. For the fresh cycles in our analysis, the daily dose did differ (200 mg three times daily of Utrogestan^®^ versus 400 mg twice daily of Cyclogest^®^). Despite the lower total dose in the Utrogestan^®^ group, there were still higher clinical pregnancy and LBRs compared to Cyclogest^®^. For the HRT-FET cycles, the dosing regimen was equivalent (400 mg twice daily for both), and the signal of benefit remained consistent with those receiving Utrogestan^®^ having higher CPRs and LBRs compared to those receiving Cyclogest^®^. Interestingly, in the HRT-FET cycles, there was also a significantly reduced MR with Utrogestan^®^; this wasn’t observed in the fresh cycles and could possibly be explained by the lower dose received in the Utrogestan^®^ group (compared to Cyclogest^®^) in the fresh cycles.

In summary, it seems prudent to develop further understanding of the absorption and mechanisms of progesterone action at an endometrial tissue level and understand individual differences based on clinical risk factors; furthermore, future randomized studies should focus on the clinical outcomes.

### Study strengths and limitations

One of the key strengths of our cohort study is the sample size; this is, by far, the largest study to date comparing vaginal oil-based progesterone to progesterone pessaries in women undergoing ART cycles. Due to the large number of variables recorded within the database, we were able to account for the majority of known confounders in the multivariable analysis, which other studies have previously failed to do. An additional strength was the focus on clinically meaningful outcomes. One of the key novel aspects of this study is that we used a dataset spanning a time period when routine serum progesterone testing was not performed, thus no rescue strategies were employed. This has allowed for a cleaner head-to-head comparison between the two forms of MVP, without the influence of rescue strategies. However, we acknowledge that having data regarding the serum progesterone levels in the HRT-FET cycles would have contributed more information and potentially influenced our understanding of the results. The absence of progesterone monitoring in our cohort introduces the possibility that a subset of patients may have had subtherapeutic progesterone levels, which could have impacted the miscarriage and live birth outcomes in the HRT-FET cycles.

The main limitation of our study is the retrospective observational nature, which means we are unable to account for residual confounding factors that may still remain and influence the outcomes. In addition, whilst we have adjusted for cause of subfertility (accounting for uterine and tubal factors) within the regression analyses, we have not explored specific subgroups of interest, such as women with adenomyosis and/or endometriosis who have known progesterone resistance.

## Conclusion

Our study indicates a higher CPR and higher LBR in all ART cycles using Utrogestan^©^ compared to Cyclogest^©^. A lower MR was also observed in the HRT-FET cycles with Utrogestan^©^ compared to Cyclogest^©^. These findings are potentially due to differences in the pharmacology of the drugs, the dosing regimen in fresh cycles, and differences in absorption amongst women with different underlying pathologies. Due to the retrospective nature of the study, residual confounding cannot be excluded. Therefore, an appropriately powered, randomized controlled trial directly comparing the two drugs, and exploring mechanisms of action in different subgroups of women with specific clinical backgrounds (e.g. endometriosis, history of recurrent pregnancy loss), is required to determine if one is superior to the other.

## Supplementary Material

deaf219_Supplementary_Figure_S1

deaf219_Supplementary_Figure_S2

deaf219_Supplementary_Table_S1

deaf219_Supplementary_Table_S2

deaf219_Supplementary_Table_S3

deaf219_Supplementary_Table_S4

deaf219_Supplementary_Table_S5

deaf219_Supplementary_Table_S6

deaf219_Supplementary_Table_S7

deaf219_Supplementary_Table_S8

## Data Availability

The data underlying this article will be shared upon reasonable request to the corresponding author. Only a fully anonymized dataset would be shared.
