# Supplementary material for: A comparison of progesterone via vaginal oil capsules versus pessaries for luteal phase support in assisted reproduction treatment: a multicentre cohort study of 42 291 cycles
Source: Hum Reprod. 2025 Nov 21;41(1):59–68. doi: 10.1093/humrep/deaf219 (PMC12769442; doi:10.1093/humrep/deaf219)
Supplement: deaf219_Supplementary_Table_S2 [file deaf219_supplementary_table_s2.pdf]

Human Reproduction, 2025, 1–1
https://doi.org/10.1093/humrep/deaf219
Supplementary Data

Supplementary Table S2. Multivariate logistic regression analyses for the effect of luteal support medications on the outcome of miscarriage.

	IVF/ICSI cycles aRR (95% CI)	HRT-FET cycles aRR (95% CI)
Age		
<35 years (ref)	———	———
35–37 years	0.99 (0.85, 1.15)	0.95 (0.87, 1.04)
38–39 years	1.01 (0.88, 1.18)	0.93 (0.85, 1.03)
40–41 years	0.98 (0.83, 1.17)	0.83 (0.74, 0.93)
≥ 42 years	0.69 (0.54.0.88)	0.87 (0.78, 0.98)
Duration of subfertility (years)	0.99 (0.97, 1.02)	1.02 (1.00, 1.04)
BMI		
<25 kg/m^2 (ref)	———	———
25–30 kg/m^2	1.10 (0.98, 1.23)	1.06 (0.98, 1.13)
>30 kg/m^2	1.22 (1.03, 1.43)	1.20 (1.08, 1.33)
Number of ART cycles	1.09 (1.05, 1.13)	1.06 (1.03, 1.09)
Ethnicity		
White (ref)	———	———
Asian	1.02 (0.86, 1.21)	0.99 (0.89, 1.11)
Black	0.75 (0.45, 1.25)	1.00 (0.80, 1.27)
Chinese	0.92 (0.56, 1.50)	1.00 (0.71, 1.44)
Mixed/Others	1.20 (0.89, 1.60)	1.06 (0.89, 1.27)
Cause of subfertility		
Ovulatory factor	1.03 (0.78, 1.37)	1.13 (1.00, 1.23)
Tubal Factor	0.75 (0.59, 0.94)	0.95(0.84, 1.09)
Uterine Factor	0.95 (0.76, 1.19)	0.87 (0.75, 1.02)
Male factor	0.87 (0.72, 1.05)	0.78 (0.69, 0.88)
Unexplained	1.05 (0.93, 1.18)	1.04 (0.96, 1.12)
Luteal support		
Cyclogest© (reference)		
Utrogestan©	1.00 (0.89 to 1.12)	0.87 (0.82 to 0.93)
AMH	0.99 (0.99, 1.00)	———
Number of oocytes	1.01 (1.00, 1.03)	———
Type of stimulation protocol		
–Long protocol (reference)		
–Antagonist protocol	1.03 (0.92, 1.16)	———
Number of embryos transferred	1.11 (0.98, 1.27)	1.18 (1.07, 1.30)
Previous live births	0.93 (0.82, 1.04)	1.02 (0.97, 1.07)
Previous miscarriages	1.10 (0.93, 1.30)	1.01 (0.91, 1.14)

aRR = adjusted Risk Ratio; HRT-FET, hormone replacement therapy-frozen embryo transfer.
