# Supplementary material for: A comparison of progesterone via vaginal oil capsules versus pessaries for luteal phase support in assisted reproduction treatment: a multicentre cohort study of 42 291 cycles
Source: Hum Reprod. 2025 Nov 21;41(1):59–68. doi: 10.1093/humrep/deaf219 (PMC12769442; doi:10.1093/humrep/deaf219)
Supplement: deaf219_Supplementary_Table_S4 [file deaf219_supplementary_table_s4.pdf]

OXFORD
human reproduction

Human Reproduction, 2025, 1–1
https://doi.org/10.1093/humrep/deaf219
Supplementary Data

Supplementary Table S4. Univariate and multivariate regression analyses for pregnancy outcomes in all cycles presenting odds ratios.

	Univariate regression OR (95% CI)	Multivariate regression Adjusted OR (95% CI)
Clinical pregnancy		
IVF/ICSI cycles	1.30 (1.22 to 1.38)	1.15 (1.02 to 1.29)‡
HRT-FET cycles	1.14 (1.08 to 1.22)	1.13 (1.06 to 1.21)#
Total miscarriage		
IVF/ICSI cycles	0.91(0.83 to 0.99)	1.03 (0.88 to 1.20)‡
HRT-FET cycles	0.85 (0.79 to 0.91)	0.84 (0.77 to 0.91)#
Early miscarriage		
IVF/ICSI cycles	0.92 (0.83 to1.02)	1.06 (0.89 to 1.27)‡
HRT-FET cycles	0.86 (0.78 to 0.93)	0.85 (0.78 to 0.93)#
Late miscarriage		
IVF/ICSI cycles	0.89 (0.77 to 1.04)	0.97 (0.75 to 1.24)‡
HRT-FET cycles	0.86 (0.75 to 0.99)	0.86 (0.73 to 0.99)#
Live birth		
IVF/ICSI cycles	1.35 (1.26 to 1.45)	1.17 (1.03 to 1.31)‡
HRT-FET cycles	1.18 (1.11 to 1.26)	1.18 (1.10 to 1.26)#

Note: Cyclogest© was used as the reference group.
‡ Logistic regression model adjusting for 12 covariates in IVF/ICSI cycles (age of female partner, BMI, AMH, number of oocytes, number of embryos transferred, cause of subfertility, ethnicity, protocol of ovarian stimulation, duration of subfertility, number of previous ART cycles, previous livebirths and previous miscarriages).
# Logistic regression model adjusting for 9 covariates in HRT-FET cycles (age of female partner, BMI, number of embryos transferred, cause of subfertility, ethnicity, duration of subfertility , number of previous ART cycles, previous livebirths and previous miscarriages).
HRT-FET, hormone replacement therapy-frozen embryo transfer; OR, odds ratio.
