# Supplementary material for: A comparison of progesterone via vaginal oil capsules versus pessaries for luteal phase support in assisted reproduction treatment: a multicentre cohort study of 42 291 cycles
Source: Hum Reprod. 2025 Nov 21;41(1):59–68. doi: 10.1093/humrep/deaf219 (PMC12769442; doi:10.1093/humrep/deaf219)
Supplement: deaf219_Supplementary_Table_S5 [file deaf219_supplementary_table_s5.pdf]

OXFORD
human reproduction
Human Reproduction, 2025, 1–1
https://doi.org/10.1093/humrep/deaf219
Supplementary Data

Supplementary Table S5. Univariable and multivariable regression analyses for pregnancy outcomes in treatment naive patients (first cycle only) comparing Cyclogest© (reference group) to Utrogestan©.

	Univariate regression RR (95% CI)	Multivariate regression Adjusted RR (95% CI)
Clinical pregnancy		
IVF/ICSI cycles	1.19 (1.13, 1.24)	1.08 (1.01, 1.15)
HRT-FET cycles	1.20 (1.13, 1.28)	1.15 (1.07, 1.23)
Total Miscarriage		
IVF/ICSI cycles	0.85 (0.74 to 0.96)	0.93 (0.76 to 1.13)
HRT-FET cycles	0.86 (0.76 to 0.98)	0.87 (0.75 to 1.00)
Early Miscarriage		
IVF/ICSI cycles	0.82 (0.70 to 0.97)	1.00 (0.78 to 1.30)
HRT-FET cycles	0.79 (0.68 to 0.92)	0.81 (0.68 to 0.95)
Late Miscarriage		
IVF/ICSI cycles	0.89 (0.71 to 1.11)	0.80 (0.57 to 1.13)
HRT-FET cycles	1.08 (0.82 to 1.41)	1.07 (0.78 to 1.46)
Live Birth		
IVF/ICSI cycles	1.22 (1.16 to 1.28)	1.11 (1.03 to 1.19)
HRT-FET cycles	1.21 (1.13 to 1.29)	1.15 (1.07 to 1.24)

HRT-FET, hormone replacement therapy-frozen embryo transfer; RR, relative risk.
