# Supplementary material for: A comparison of progesterone via vaginal oil capsules versus pessaries for luteal phase support in assisted reproduction treatment: a multicentre cohort study of 42 291 cycles
Source: Hum Reprod. 2025 Nov 21;41(1):59–68. doi: 10.1093/humrep/deaf219 (PMC12769442; doi:10.1093/humrep/deaf219)
Supplement: deaf219_Supplementary_Table_S6 [file deaf219_supplementary_table_s6.pdf]

Human Reproduction, 2025, 1–1
https://doi.org/10.1093/humrep/deaf219
Supplementary Data

Supplementary Table S6. Univariate and multivariate regression analyses for pregnancy outcomes in all cycles with singleton pregnancies comparing Cyclogest© (reference group) to Utrogestan©.

	Univariate regression RR (95% CI)	Multivariate regression Adjusted RR (95% CI)
Clinical pregnancy		
IVF/ICSI cycles	1.19 (1.13 to 1.24)	1.08 (1.02 to 1.15)
HRT-FET cycles	1.08 (1.04 to 1.12)	1.06 (1.02 to 1.11)
Total miscarriage		
IVF/ICSI cycles	0.92 (0.85 to 0.99)	1.00 (0.90 to 1.12)
HRT-FET cycles	0.88 (0.82 to 0.93)	0.87 (0.82 to 0.93)
Early miscarriage		
IVF/ICSI cycles	0.93 (0.85 to 1.03)	1.05 (0.91 to 1.21)
HRT-FET cycles	0.88 (0.82 to 0.94)	0.87 (0.80 to 0.94)
Late miscarriage		
IVF/ICSI cycles	0.90 (0.78 to 1.04)	0.91 (0.74 to 1.12)
HRT-FET cycles	0.87 (0.76 to 0.99)	0.89 (0.77 to 1.03)
Live birth		
IVF/ICSI cycles	1.24 (1.18 to 1.30)	1.11 (1.04 to 1.19)
HRT-FET cycles	1.11 (1.07 to 1.16)	1.09 (1.04 to 1.14)

RR, relative risk; RT-FET, hormone replacement therapy-frozen embryo transfer.
