# Supplementary material for: A comparison of progesterone via vaginal oil capsules versus pessaries for luteal phase support in assisted reproduction treatment: a multicentre cohort study of 42 291 cycles
Source: Hum Reprod. 2025 Nov 21;41(1):59–68. doi: 10.1093/humrep/deaf219 (PMC12769442; doi:10.1093/humrep/deaf219)
Supplement: deaf219_Supplementary_Table_S7 [file deaf219_supplementary_table_s7.pdf]

OXFORD
human reproduction
Human Reproduction, 2025, 1–1
https://doi.org/10.1093/humrep/deaf219
Supplementary Data

Supplementary Table S7. Univariate and multivariate regression analyses for pregnancy outcomes in all cycles comparing Cyclogest© (reference group) to Utrogestan© with multiple imputations for missing data.

	Univariate regression RR (95% CI)	Multivariate regression Adjusted RR (95% CI)
Clinical pregnancy		
IVF/ICSI cycles	1.18 (1.13 to 1.24)	1.05 (1.01 to 1.10)
HRT-FET cycles	1.09 (1.05 to 1.13)	1.08 (1.04 to 1.12)
Total miscarriage		
IVF/ICSI cycles	0.92 (0.85 to 0.99)	0.94 (0.87 to 1.03)
HRT-FET cycles	0.87 (0.82 to 0.93)	0.87 (0.82 to 0.93)
Early miscarriage		
IVF/ICSI cycles	0.93 (0.84 to1.02)	0.98 (0.88 to 1.09)
HRT-FET cycles	0.87 (0.81 to 0.94)	0.86 (0.80 to 0.93)
Late miscarriage		
IVF/ICSI cycles	0.89 (0.78 to 1.03)	0.88 (0.75 to 1.02)
HRT-FET cycles	0.87 (0.76 to 0.99)	0.90 (0.78 to 1.03)
Live birth		
IVF/ICSI cycles	1.15 (1.10 to 1.20)	1.05 (1.01 to 1.10)
HRT-FET cycles	1.11 (1.07 to 1.16)	1.10 (1.06 to 1.15)

HRT-FET, hormone replacement therapy-frozen embryo transfer; RR, relative risk.
