# Supplementary material for: A comparison of progesterone via vaginal oil capsules versus pessaries for luteal phase support in assisted reproduction treatment: a multicentre cohort study of 42 291 cycles
Source: Hum Reprod. 2025 Nov 21;41(1):59–68. doi: 10.1093/humrep/deaf219 (PMC12769442; doi:10.1093/humrep/deaf219)
Supplement: deaf219_Supplementary_Table_S8 [file deaf219_supplementary_table_s8.pdf]

Human Reproduction, 2025, 1–1
https://doi.org/10.1093/humrep/deaf219
Supplementary Data

Supplementary Table S8. Univariate and multivariate regression analyses after propensity matching for pregnancy outcomes in all cycles comparing Cyclogest© (reference group) to Utrogestan© with multilevel regression analysis using ethnicity as level II variable.

	Univariate regression RR(95% CI)	Multivariate regression Adjusted RR (95% CI)
Clinical pregnancy		
IVF/ICSI cycles	1.19 (1.12 to 1.25)	1.08 (1.00 to 1.17)
HRT-FET cycles	1.09 (1.04 to 1.14)	1.08 (1.03 to 1.13)
Total miscarriage		
IVF/ICSI cycles	0.92 (0.85 to 0.99)	0.97 (0.86 to 1.10)
HRT-FET cycles	0.87 (0.82 to 0.94)	0.88 (0.82 to 0.94)
Early miscarriage		
IVF/ICSI cycles	0.93 (0.84 to 1.03)	1.02 (0.88 to 1.18)
HRT-FET cycles	0.87 (0.81 to 0.95)	0.87 (0.80 to 0.94)
Late miscarriage		
IVF/ICSI cycles	0.90 (0.78 to 1.04)	0.89 (0.72 to 1.10)
HRT-FET cycles	0.87 (0.76 to 1.00)	0.90 (0.78 to 1.04)
Live birth		
IVF/ICSI cycles	1.24 (1.17 to 1.31)	1.12 (1.03 to 1.22)
HRT-FET cycles	1.12 (1.07 to 1.18)	1.10 (1.05 to 1.16)

HRT-FET, hormone replacement therapy-frozen embryo transfer; RR, relative risk.

© The Author(s) 2025. Published by Oxford University Press on behalf of European Society of Human Reproduction and Embryology.
This is an Open Access article distributed under the terms of the Creative Commons Attribution License (https://creativecommons.org/licenses/by/4.0/), which permits unrestricted reuse, distribution, and reproduction in any medium, provided the original work is properly cited.
Human Reproduction, 2025, 00, 1–20
https://doi.org/10.1093/humrep/deaf219
Original Article
